# Hindering of Cariogenic *Streptococcus mutans* Biofilm by Fatty Acid Array Derived from an Endophytic *Arthrographis kalrae* Strain

**DOI:** 10.3390/biom10050811

**Published:** 2020-05-25

**Authors:** Marwa M. Abdel-Aziz, Tamer M.Emam, Marwa M. Raafat

**Affiliations:** 1 Regional Center for Mycology and Biotechnology (RCMB), Al-Azhar University, Cairo 11651, Egypt; marwa2rcmb@yahoo.com; 2 Microbiology Department, Desert Research Center (DRC), Cairo 11753, Egypt; tameremam7@gmail.com; 3 Microbiology and Immunology Department, Faculty of Pharmaceutical Sciences and Pharmaceutical Industries, Future University in Egypt (FUE), Cairo 11835, Egypt

**Keywords:** Endophytic fungi, endophytic fatty acids, *Coriandrum sativum*, antibiofilm, extracellular polysaccharides, *Streptococcus mutans* virulence, oral biofilm

## Abstract

*Streptococcus mutans* has been considered as the major etiological agent of dental caries, mostly due to its arsenal of virulence factors, including strong biofilm formation, exopolysaccharides production, and high acid production. Here, we present the antivirulence activity of fatty acids derived from the endophytic fungus *Arthrographis kalrae* isolated from *Coriandrum sativum* against *Streptococcus mutans*. The chemical composition of the fatty acids was analyzed by gas chromatography–mass spectrometry GC-MS and revealed nine compounds representing 99.6% of fatty acids, where unsaturated and saturated fatty acids formed 93.8% and 5.8 % respectively. Oleic and linoleic acids were the major unsaturated fatty acids. Noteworthy, the fatty acids at the concentration of 31.3 mg L^–1^ completely inhibited *Streptococcus mutans* biofilm, and water insoluble extracellular polysaccharide production in both polystyrene plates, and tooth model assay using saliva-coated hydroxyapatite discs. Inhibition of biofilm correlated significantly and positively with the inhibition of water insoluble extracellular polysaccharide (R = 1, *p* < 0.0001). Furthermore, *Arthrographis kalrae* fatty acids at a concentration of 7.8 mg L^–1^ exhibited acidogenesis-mitigation activity. They did not show bactericidal activity against *Streptococcus mutans* and cytotoxic activity against human oral fibroblast cells at the concentration used. On the other hand, saliva-coated hydroxyapatite discs treated with sub-minimum biofilm inhibitory concentration of fatty acids showed disturbed biofilm architecture with a few unequally distributed clumped matrices using fluorescence microscopy. Our findings revealed that the intracellular fatty acid arrays derived from endophytic *Arthrographis kalrae* could contribute to the biofilm-preventing alternatives, specifically *Streptococcus mutans* biofilms.

## 1. Introduction

Dental caries is a predominant worldwide oral disease affecting almost half of the world’s population [1]. In spite of all the public health efforts focusing on the importance of oral hygiene and dental health, dental caries is a major problem in adults and children that results in tooth loss especially in children [2]. Dental caries results from establishment of cariogenic biofilms on tooth enamel. The formation of oral biofilm starts by the adsorption of saliva on the tooth enamel resulting in the formation of a complex mixture of glycoproteins, acidic proline–rich proteins, mucins, bacterial cell debris, exoproducts, and sialic acid. This is followed by a primary reversible sucrose–independent bacterial interaction with the complex components. Finally, more bacterial strains adhere irreversibly with the formation of extracellular polysaccharides (EPSs) mediated by sucrose fermentation products (such as glucans and fructans) in combination with glucan-binding proteins resulting in dental plaque biofilm formation [3,4,5]. Carbohydrate rich diets, particularly sucrose, play an important role in the development of dental caries as they increase acid production that surpasses both the capacity of the saliva to clear all the acid end-products and the neutralization activity of the salivary/plaque buffer system, which results in more frequent acidification of the plaque [6].

Among the numerous bacteria associated with dental caries pathogenesis, the main cariogenic microbe is *Streptococcus mutans* (*S. mutans*) that inseparably relies on a set of virulence factors to establish cariogenic infection by formation of a strong biofilm. These factors include initial adherence to the tooth through high-affinity adhesins, accumulation and persistence of bacteria due to EPS production, high rate acid production attributable to its higher ability to metabolize sucrose in addition to its acid resistance and ability to grow at low pH [5,7,8,9]. The strong biofilms formed by *S. mutans* on a tooth’s surface plays a critical role in dental plaque and dental cavity formation [10]. Furthermore, *S. mutans* utilize dietary sucrose and secrete glucosyltransferases and fructosyltransferase to synthesize EPS, especially water-insoluble glucans. EPS contributes to biofilm adherence, stability, and integrity [7]. It also acts as a scaffold for other oral pathogens to colonize and form dental plaque [11].

Prevention of dental plaque can be achieved by many antimicrobial products that inhibit the growth of cariogenic oral pathogens, however, they have the disadvantage of their short-term efficacy [3,12]. Furthermore, normal bacterial flora in the body is inhibited by the frequent use of wide-spectrum antimicrobial agents, which also have the potential to cause serious adverse effects [13]. Therefore, exploring novel agents that have the ability to target the virulence factors of *S. mutans* contributing to cariogenic biofilm formation without suppressing the oral microbial residents is a global demand.

Coriander (*Coriandrum sativum* L.) is an aromatic annual herb and is characterized by essential oil and fatty acid contents. It is commonly used in food, aromatherapy, medicine, and pharmaceutical industries [14]. Endophytic fungi are fungi that colonize plants and can asymptomatically thrive in the plant tissues such as leaves, stems, and roots [15]. Endophytic fungi play beneficial roles for the plant host such as enhancing host resistance to insect feeding; enhancing the ecophysiology of the plants, thus enabling them to counter abiotic stresses such as drought; and increasing plant biomass. These benefits are principally attributed to endophytic secondary metabolites [16,17]. Hence, endophytic fungi are very promising biological resources, which act as endless sources of bioactive compounds for many pharmaceutical and medical applications. Huge diversity in endophytic microorganisms implies a broad diversity of biologically active metabolites with different biological categories such as antitumor, anti-diabetic, immunemodulatory, herbicidal, antifungal, antiviral, and antibacterial [18,19]. Previous studies have reported numerous biological properties of *Coriandrum sativum* L., including antioxidant, antimicrobial, hypoglycemic, antianxiety, antihyperlipidemic, anti-inflammatory, analgesic, anti-convulsant, and anti-cancer activities [14]. In spite of that, occasional studies have focused on the endophytes from *Coriandrum sativum,* Recently, Pundir et al. (2018) and Beiranvand et al. (2017) reported isolation of endophytic bacteria from *Coriandrum sativum’s* root and leaves, respectively [19,20]. However, to the best of our knowledge, no previous reports investigated fungal endophytes from *Coriandrum sativum*. On the other hand, a few endophytic fungal bioactive metabolites with antibiofilm activity have been reported [21]. For example, the extracellular metabolites of an endophytic *Aspergillus nidulans* strain showed potent antibiofilm activities against oral pathogenic strains of *Candida albicans* [22]. Also, extracellular metabolites of an endophytic *Eurotium chevalieri* isolate revealed potent broad spectrum antibacterial activity and inhibition of bacterial biofilms [21]. Based on the particular attention drawn on the biological activities of endophytic metabolites—specially, antimicrobial lipids in numerous studies [23,24,25,26]—the present study aimed at extracting fatty acids from an endophytic fungus isolated from *Corandium sativium* leaves and investigate the antivirulence activity of the extracted fatty acids combination against *S. mutans*.

## 2. Material and Methods

### 2.1. Isolation of Endophytic Fungus from the Collected Plant Samples 

Samples of *Corandium sativium* plant were randomly collected from the south Elqantara Sharq regional station, Desert Research Center (DRC), Sinai, Egypt. The healthy leaves of the plant were rinsed with water. In order to surface sterilize the leaves; they were immersed for 1 min in 70% ethanol, followed by 3 min in 2% sodium hypochlorite. The sterilized leaves were washed five times with sterilized water, allowed to dry under aseptic conditions, and cut into small parts (about 10 mm length). The dried sterilized fragments were cultured on potato dextrose agar (PDA) (Hi-media, India) plates augmented with 200 µg mL^−1^ chloramphenicol, followed by incubation of the plates for ten days at 25 °C [27]. The plates were checked daily for any fungal growth. The growing out fungal hyphea were isolated and purified by subsequent culturing in new PDA plates. Pure fungus was cultured on PDA slants and preserved at 4 °C [28].

### 2.2. Selection and Identification of an Endophytic Isolate

Nile red staining assay was used in order to examine the presence of intracellular lipid content. Briefly, 2–3 × 10^4^ CFU mL^−1^ of fungal isolates were incubated with 0.05 mL Nile-red solution (25 µg Nile-red; Sigma Aldrich, Germany / L of acetone) at 37 °C for 30 min [29,30]. The stained lipid molecules were visualized using fluorescence microscope (FM; LEICA. Wetzlar, Germany) equipped with a DFC camera at excitation and emission wavelengths 530 nm and 635 nm, respectively. Then the endophytic isolate was identified using morphological and microscopical characteristics after staining with lactophenol cotton blue. Furthermore, molecular identification of the endophytic isolate, was confirmed based on internal transcribed spacer (ITS)-rDNA sequencing utilizing the fungal universal primer set of ITS1 (5′-TCC GTA GGT GAA CCT GCG G-3′) and ITS4 (5′-TCC TCC GCT TAT TGA TAT GC-3′) [31]. PCR thermal cycling conditions were as follows: initial denaturation at 95 °C for 10 min, denaturation at 95 °C for 30 s, annealing at 57 °C for 1 min, extension at 72 °C for 1 min for 35 cycles, and a final extension at 72 °C for 10 min [32]. Purification of the PCR product was done using the PCR purification kit (Qiagen, Germany) according to the manufacturer’s instructions. Purified PCR products were sequenced using an automatic sequencer (ABI Prism 377; Applied Biosystems, CA, USA). NCBI-BLAST was used to identify sequence homologues [33].

### 2.3. Extraction and Analysis of the Fatty Acids from the Identified Endophytic Isolate

The identified endophytic fungal isolate was grown in potato dextrose broth media (PDB, Hi-media, India) on a rotary shaker (150 rpm) at 25 °C for six days, followed by mycelia collection and lyophilization. Fatty acid extraction was performed according to Grantina-Ievina et al. (2014) [34]. Briefly, 100 mg of lyophilized fungal biomass was subjected to supercritical fluid extractor (ISCO 220, USA) equipped with a syringe pump (260 D), with 1.4 mg/hour of CO_2_, at 80 °C under 320 atm pressure. The analysis of the fatty acids fraction was carried out using a gas chromatograph coupled to a mass spectrometer (GC-MS; Shimadzu QP2010, Japan) [35]. A GC-capillary column ZB5MS was used with the following dimensions: 30 m × 0.25 mm × 0.25 µm film thickness. The sample was diluted, and about 1 µL was injected manually with splitless mode. The flow rate of the helium carrier gas was 1 mL/min, and the temperature was initiated by 60 °C and then raised to 240 °C at a rate of 3 °C/min. The injector and detector temperatures were adjusted at 230 °C. The MS ionization of the molecules was performed by electron impact ionization (EI) mode at 70 eV with a 50–500 m/z scan mode range of the mass detector. The temperature of the ion source was 200 °C, while the quadrupole temperature was 250 °C. The percentage of composition of the tentatively identified fatty acids was computed and calculated relatively from the GC peaks areas without any correction factors. Total fatty acids were calculated as the sum of saturated and unsaturated fatty acid content. Fatty acids were identified by comparative analysis of their spectral fragmentation pattern with those of the WILEY/NIST mass spectral database [36,37].

### 2.4. Antivirulence Activity of Arthrographis kalrae Fatty Acids (AKFAs) against S. mutans

#### 2.4.1. Antibiofilm Activity

Antibiofilm activity of *Arthrographis kalrae* fatty acids (AKFAs) was evaluated against *S. mutans* ATCC 25175 biofilm using static crystal violet microtiter plate assay as reported by Merritt et al. (2011) [38]. First, *S. mutans* ATCC 25175 was cultured in brain heart infusion broth (Hi-media, India) including 1% sucrose (BHI-S) overnight at 37 °C. After incubation, the inoculums were adjusted at a concentration of 1–2× 10^6^ CFU mL^−1^. About 20 µL of the adjusted bacterial suspension was inoculated into a sterile microtiter tissue culture plate containing 180 µL fresh BHI-S with two-fold serially diluted AKFAs to achieve concentrations ranging from 125 mg L^−1^ to 1 mg L^−1^ in 5% DMSO into triplicate wells. The addition of 5% Dimethyl sulfoxide (DMSO) instead of AKFAs in triplicate wells was included to serve as a positive control, while non-inoculated growth medium served as negative control. All the plates were incubated in a 5% CO_2_ incubator overnight at 37 °C. After incubation, free losing cells were gently discarded, and the microtiter wells were washed with sterile phosphate buffer saline (PBS) three times and then allowed to dry for 10 min. The adherent biofilm cells were stained by adding 100 µL of 0.1% crystal violet to each well. After 15 min, excess stain was removed, and the wells were rinsed three times with water and air dried. The stain bound to the biofilm cells were resuspended in 30% acetic acid for 10 min. The absorbance was measured at 590 nm by a microplate reader (Biotik, USA). The percent of biofilm inhibition was determined using the following equation: Inhibition of biofilm formation (%) = [1 − (OD_590_ treated / OD_590_ positive control)] × 100 [39].The biofilm inhibition assay was repeated twice with three replicates in each. The minimum biofilm inhibitory concentration (MBIC), which is the lowest concentration that showed complete biofilm prevention, was detected. The MBIC_50_ was estimated using GraphPad Prism software.

#### 2.4.2. Inhibition of S. mutans Biofilm’s Water Insoluble EPS Assay

*S. mutans* biofilms were prepared and treated with AKFAs concentrations as described above. Planktonic cells were discarded, and biofilms were washed three times with sterile PBS to ensure the removal of all non-adherent cells. One milliliter of sterile PBS was added to each well, and the content of the wells was vigorous shaken using pipette and vortex. The biofilm cell suspensions were collected in sterile microcentrifuge tubes. Detachment of the extracellular matrix from the cells was performed by sonication of the biofilm cells on ice for 3 × 30 s cycles [40]. The biofilm suspension was centrifuged at 8700× *g* for 10 min at 4 °C, the supernatant was gently removed, and the pellets washed five times with sterile PBS for complete removal of all water-soluble EPS. Next, 1.0 M NaOH was added and incubated for 2 h at 37 °C with agitation to extract the water insoluble EPS (WI-EPS) [10]. The amount of WI-EPS was determined using the anthrone-sulfuric acid method. Three volumes of freshly prepared anthrone-sulfuric acid reagent (0.1 g of anthrone; Sigma Aldrich, Germany / 100 mL of concentrated sulfuric acid) were added to the sample and heated in a water bath at 95 °C for 5 min. The sample was cooled down to room temperature and read at an absorbance of 625 nm. The assay was conducted twice each in triplicate. The percentage of WI-EPS inhibition was calculated as follows: Inhibition of WI-EPS production (%) = [1 − (OD_625_ treated / OD_625_ positive control)] × 100 [10]. The lowest concentration that prevents WI-EPS production completely was determined as minimum WI-EPS inhibitory concentration (MIC_WI-EPS_). The MIC50_WI-EPS_ was estimated using GraphPad Prism.

#### 2.4.3. Acidogenesis- Mitigating Assay

Treatment of *S. mutans* biofilms with different concentrations of AKFAs was repeated, as described above, to determine the degree of acidification of the growth media. After overnight incubation of the microtiter plates in 5% CO_2_ at 37 °C, 10 μl of phenol red solution was added directly to each well, incubated for 15 min at room temperature, and then examined for color change. Positive controls were *S. mutans* with 5% DMSO in growth medium, while negative controls were non-inoculated wells containing the equivalent concentration of AKFAs. The assay was repeated three times, and the minimum acid neutralizing concentrations (MNC), which is the lowest concentration, showed no change in the phenol red color indictor was detected [10].

### 2.5. Bacterial Viability Assay 

To evaluate the effect of AKFAs on the viability of *S. mutans,* XTT (2,3-bis (2 methoxy-4-nitro-5-sulfophenyl)-5-[(phenylamino)-carbonyl]-2H-tetrazolium hydroxide) reduction assay was conducted two times [11]. First, formation of *S. mutans* biofilm in the absence and presence of AKFAs (1000–2 mg L^−1^) was performed twice as previously described in this study using 12-well microtiter plates. Planktonic and biofilm cells were collected and transferred to a new sterile microtiter plate by scratching the wells with a pipette tip. To ensure the transfer of all cells, 500 μl of PBS was added to the each well and transferred again to the same plate. Planktonic cells of the second microtiter plate were collected, carefully separated, and transferred to another sterile microtiter plate. The biofilm cells in the second plate were resuspended with 200 μl of PBS and transferred to a third sterile microtiter plate. Another 500 μl of PBS was added again to recover any remaining adherent cells. One hundred microliters of freshly prepared XTT solution (0.5 g of XTT (Sigma Aldrich, Germany) in 1 L PBS) was added into every well (planktonic, biofilm, and total cells) and incubated at 25 °C for 2 h in the dark. The colorimetric change was measured using microtiter plate reader at 492 nm [41]. 

### 2.6. Antivirulence Activity of AKFAs on S. mutans Biofilm Using an In Vitro Tooth Model

#### 2.6.1. Saliva-Coated Hydroxyapatite (S-H) Disc Assay

The saliva-coated hydroxyapatite (S-H) disc procedure was conducted as previously described by Lemos et al. (2010) using sterile hydroxyapatite discs (3D Biotek, USA) [42]. In a sterile tube containing 10 mL of clarified saliva, 10-mm hydroxyapatite discs were combined and vortexed to increase coating of the discs. A six-well culture plate was inoculated with 1 mL of 3–5 × 10^6^ of *S. mutans* in BHI-S and treated at the MBIC and MBIC50 of AKFAs in the first and second rows, respectively. One S-H disc pre-coated with saliva was submerged vertically in each well and then incubated for 24 h at 37 °C, 5% CO_2_. Plates were also inoculated with *S. mutans* in 5% DMSO (positive control) and non–*S. mutans* containing wells (negative controls). After incubation, discs that previously treated with AKFAs MBIC were removed, washed three times with sterile 0.9% NaCl, and placed in glass tubes containing 1 mL 0.9% NaCl. Then, all the discs were subjected to 10 min ultrasonication bath, followed by sonication (2 × 5 pulses) to split up cell aggregates. Cells were serially diluted in PBS, cultured on mitis salivarius bacitracin agar (Hi-media, India) plates and incubated at 37 °C for 48–72 h in a 5% CO_2_ incubator. After incubation, colonies were counted, and total count of cells was calculated and expressed as CFU/disc. For determination of the pH, after incubation with AKFAs growth media were examined for acid production by adding 10 μl of phenol red solution directly to each well, followed by the detection of any color changes after 15 min. The S-H disc assay was conducted twice in three replicates. 

#### 2.6.2. Microscopic Observations of S-H Discs Treated with MBIC_50_ of AKFAs

S-H discs treated with MBIC_50_ of AKFAs were visualized by scanning electron microscopy (SEM) to detect their effect on biofilm structure and fluorescence microscopy (FM) to investigate their effect on EPS matrix [43]. The discs without AKFAs were used as a control. For SEM preparation, discs were fixed by treatment with 3% glutaraldehyde for 2 h, followed by post-fixation using 1% osmium tetroxide for 1 h at room temperature. Dehydration of the discs were done using graded ethanol series from 10 to absolute ethanol (15 min each), and then a drying critical point device (EMS 850, USA) were used to dry the discs up to the critical point with CO_2_. The dried discs were covered with gold using an SPI Module TM Sputter Coater and examined using a JEOL JSM-5500LV at 20 kV accelerating voltage [44]. For FM preparation, EPS were stained with green-fluorescent Alexa Fluor 488 conjugate of succinylated concanavalin A and incubated in the dark at 25 °C for 2 h. Observation of WI-EPS bind to concanavalin A that emits green fluorescence was done using a LEICA microsystem (Wetzlar Microscope GmbH, DM5000 B, Germany) [45].

### 2.7. Oral Fibroblast Viability Assay 

The cytotoxicity of AKFAs to oral fibroblast cells was estimated by investigating the effect of AKFAs on human embryonic palatal mesenchyme (HEPM; ATCC CRL-1486) cells, briefly; flat- bottom 96-well plates were inoculated with HEPM cells grown in Eagle’s minimum essential medium (EMEM; Sigma Aldrich, Germany) augmented with 10% fetal bovine serum (FBS), 100 μg/mL streptomycin, and 100 U/mL penicillin and incubated overnight at 37 °C, 5% CO_2_. After the medium was removed, fresh EMEM medium containing 2% FBS were added, and two-fold serial dilution of AKFAs achieved concentrations ranging from 125 to 1 mgL^−1^, was performed. Plates were incubated at 37 °C for 24 h. After incubation, medium was discarded, and 100 µL of PBS and 100 µL Cell Titer-Glo 2.0 reagent (Promega, USA) were added. The microtiter plates were re-incubated for 10 min at 37 °C. The absorbance was measured using a microtiter plate reader at 450 nm. The cytotoxicity assay repeated three times. The percentage of inhibition was determined as follow: [100 − (sample OD_450_ − blank OD_450_)/( control OD_450_ − blank OD_450_) × 100%] [46].

### 2.8. Statistical Analysis 

Statistical analysis was performed using GraphPad Prism version 6.0 software (GraphPad Software, Inc., La Jolla, CA, USA). Student’s t-test was used to calculate the statistical significance. Experiments values were presented as mean and standard deviation (SD) and 95% confidence interval values. The level of significance was set at *p* < 0.05.

## 3. Results

In the present study, endophytic fungus was isolated from healthy *Corandium sativium* leaves. Total fatty acids produced by this endophytic fungus were extracted, identified, and examined for their *S. mutans* antivirulence activities.

### 3.1. Isolation and Identification of the Selected Endophytic Isolate

**Figure 1:**
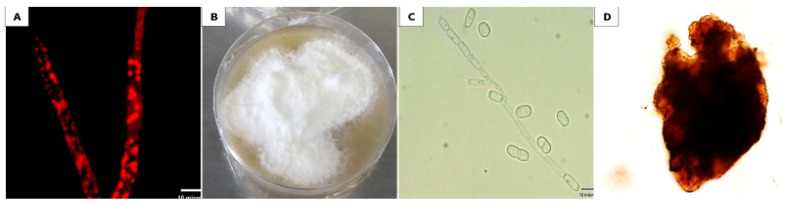
Microscopic and macroscopic features of *Arthrographis kalrae*. (**A**) Fluorescence microscopic observation of *Arthrographis kalrae* hyphae stained with Nile red stain marked with intracellular fluorescent red lipid globules. (**B**) Morphological appearance of *Arthrographis kalrae.* (**C** )Asexual morph with poorly branched conidiophore bearing arthroconidia and lateral sessile conidia. (**D**) Sexual morph with dark brown hyphae on the peridium.

The endophytic fungus that was isolated from the leaves of the plant host *Corandium sativium* showed markedly red lipid globules using FM after staining with Nile red stain (Figure 1A). 

Morphological examination of the isolate revealed white, profuse aerial mycelia with irregular margins, and a brownish reverse side (Figure 1B). Microscopically, the fungus was found to be able to produce both asexual arthroconidia (Figure 1C) and sexual ascospores (Figure 1D). Furthermore, molecular identification was done by comparing the obtained sequence with other sequences in GenBank database using NCBI BLAST [33]. Accordingly, the ITS rDNA sequence showed a 96.8 % homology to *Arthrographis kalrae.* The sequence was submitted to GenBank with accession number MK967698.

### 3.2. Chemical Composition of Arthrographis kalrae’s AKFAs 

**Figure 2:**
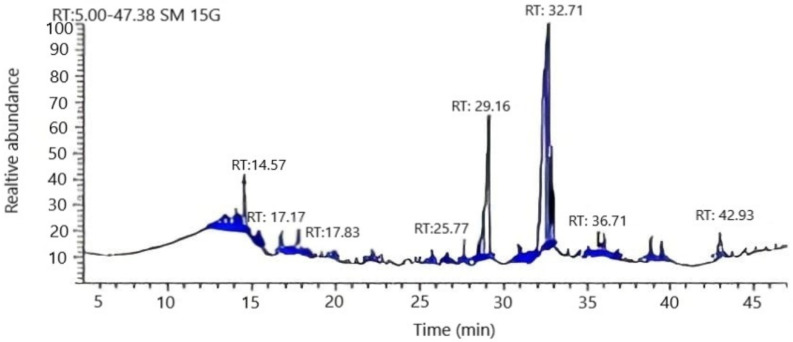
Gas chromatograms of intracellular fatty acid composition of the endophytic *Arthrographis kalrae* isolate.

Nine compounds were identified by GC-MS analysis representing 99.6% of *Arthrographis kalrae* fatty acids (Figure 2). The detected fatty acids were categorized into two groups—unsaturated fatty acids representing 93.8%, and 5.8% saturated fatty acids. Moreover, the major components of unsaturated fatty acids were oleic and linolenic acids representing 61% and 23.9%, respectively. Interestingly, docosahexaenoic acid, an omega 3 fatty acid, was found to constitute 4.6% of the unsaturated fatty acids (Table 1).

### 3.3. Antivirulence Activity of the Extracted AKFAs on S. mutans

**Figure 3:**
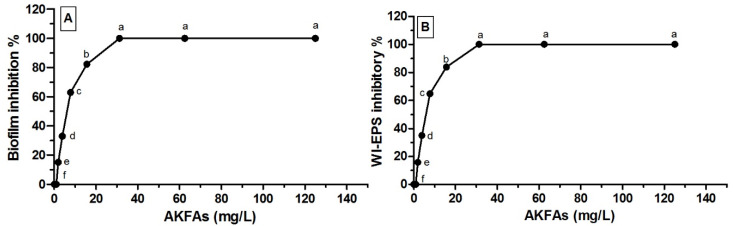
Antivirulence activity of *Arthrographis kalrae* fatty acids (AKFAs). (**A**) AKFAs inhibitory activity against *S. mutans* biofilm formation in (*p* < 0.0001). (**B**) WI-EPS inhibitory activity of AKFAs against *S. mutans* (*p* < 0.0001). Different letters indicate significant differences between AKFAs concentrations according to Tukey post hoc test at *p* < 0.05 in both (**A**) and (**B**).

**Figure 4:**
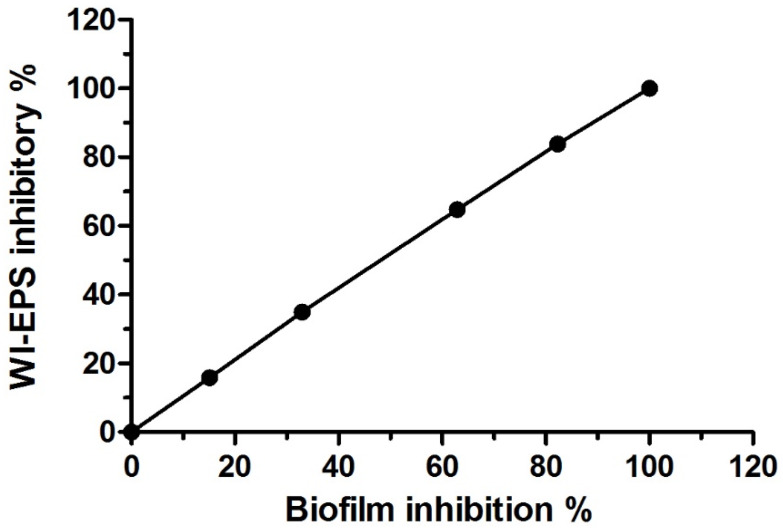
Scatter plot showing correlation between mean biofilm and WI-EPS inhibitory percentage at different AKFA concentrations (R = 1, *p* < 0.0001).

The AKFAs of *Arthrographis kalrae* was found to exhibit significant inhibitory effects on both biofilm formation and WI-EPS production of *S. mutans* (*p* < 0.05) in a dose-related way as the percentage of biofilm and WI-EPS inhibition is in a direct relationship with AKFAs concentrations. The percent of inhibition for both parameters reached 100% at 31.3, 62.5 and 125 mg L^−1^ (Table 2). The AKFAs were able to completely inhibit the *S. mutans* biofilm and WI-EPS matrix production at a concentration of 31.3 mg L^−1^. Accordingly, this concentration (31.3 mg L^−1^) was considered as the MBIC as well as MIC_WI-EPS_ (Figure 3A,B). Both MBIC_50_ and MIC_50 WI-EPS_ were estimated as the concentrations that inhibit 50% of the biofilm formation and WI-EPS production, respectively, using Graphprism (Table 2). Furthermore, the results revealed a positive correlation between inhibition of biofilm formation and WI-EPS production (R = 1, *p* < 0.0001) (Figure 4).

**Figure 5:**
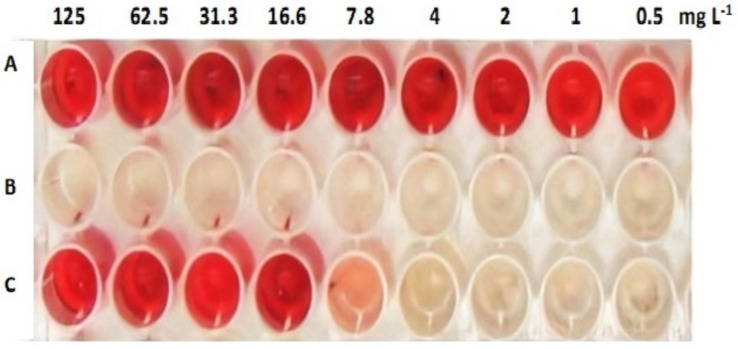
Acid mitigation effect of AKFAs on *S. mutans* biofilm detected by phenol red indicator. Row A: AKFAs concentrations in the medium (no color change in phenol red indicates no acid production). Row B: Untreated *S. mutans* cells (*S. mutans* grow with acid production indicated by pale yellow color of phenol red). Row C: *S. mutans* treated with AKFAs showed acidogensis inhibition effect by 125–7.8 mg L^−1^ concentrations (red color of phenol red indicator in the first five wells) while AKFAs concentrations less than 7.8 mg L^−1^ showed diminished acidogensis inhibition indicated by pale yellow in the last four wells, MNC = 7.8 mg L^−1^).

The inhibition of *S. mutans* acidogenesis by AKFAs was assessed in 96-well plates. It was found that incubating AKFAs with *S. mutans* cells revealed acid neutralizing activity with an MNC = 7.8 mg L^−1^ (Figure 5). However, no color change was observed in wells containing AKFAs of varying concentrations. Despite the efficient inhibitory activity of AKFAs on biofilm formation and WI-EPS production, AKFAs showed no bactericidal activity against *S. mutans* planktonic cells at 1000–2 mg L^−1^ using the sensitive XTT assay.

### 3.4. Activity of AKFAs on S. mutans Biofilm Using an In Vitro Tooth Model

**Figure 6:**
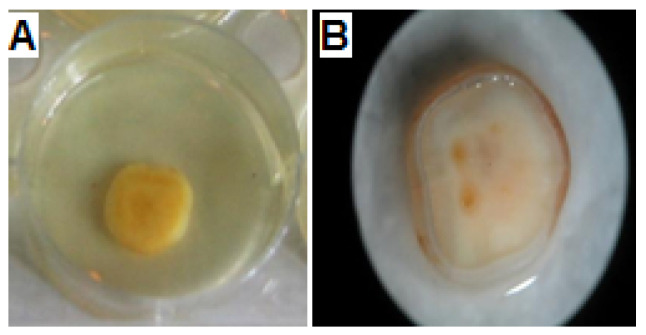
In vitro tooth model assay using saliva-coated hydroxyapatite (S-H) discs. (**A**) Saliva-coated S-H disc in a well of a six-well plate inoculated with *S. mutans* and treated with MBIC of AKFAs. (**B**) Stereographic image of *S. mutans* biofilm formed on saliva coated S-H disc.

An established in vitro tooth model that simulates tooth enamel coated with a salivary layer was used to examine the effect of AKFAs on *S. mutans* biofilm. The saliva coated SH discs were incubated with *S. mutans* for 24 h and the MBIC of AKFAs was determined. (Figure 6A). After incubation, attached biofilm cells to the SH discs (Figure 6B) were collected, detached by ultasonication, inoculated on mitis salivarius bacitracin agar plates, and then counted. Complete inhibition of biofilm cells that was previously treated with the MBIC of AKFAs (31.3 mg L^−1^) per disc was observed (zero CFU/mL) compared to the untreated control *S. mutans* biofilm cells (2.6 × 10^6^ CFU/mL) (Figure S1). The MBIC of AKFAs was also found to reduce the acidogensis production of *S. mutans* biofilm cells on SH discs, as indicated by the phenol red indicator (Figure S2).

**Figure 7:**
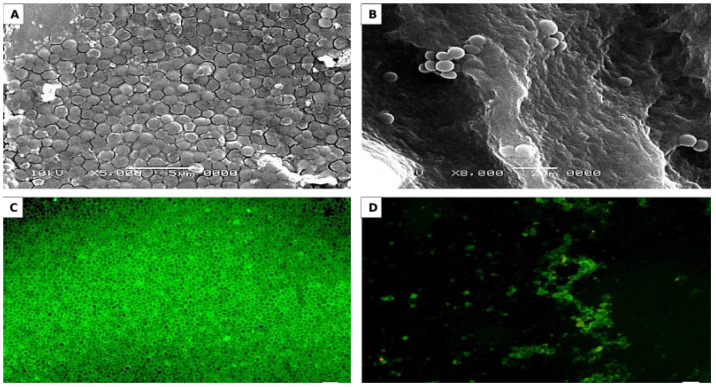
Microscopic examination of *S. mutans* biofilm formed on saliva coated S-H disc and treated with MBIC_50_ of AKFAs. (**A**,**B**) Scanning electron microscopy observation of untreated and treated *S. mutans* biofilm cells with MBIC_50,_ respectively. (**C**,**D**) Flourescent microscopy observation of untreated *S. mutans* matrix and *S. mutans* matrix treated with MBIC_50_ of AKFAs, respectively.

MBIC_50_ of AKFAs was used for studying the alteration in *S. mutans* biofilm structure that formed on SH discs using SEM to detect the changes in biofilm architecture and the morphological appearance of the cells. The SEM micrographs revealed that the *S. mutans* biofilm without any treatment appeared uniform and firmly attached with high structural arrangement (Figure 7A). In contrast, the disc treated with MBIC_50_ of AKFAs showed a deleterious effect on biofilm morphology with few irregular clumps of cells adhering to the disc surface (Figure 7B).

Additionally, the effect of MBIC_50_ of AKFAs on the EPS matrix of the biofilm was investigated by staining the discs with a fluorescent EPS-binding dye followed by visualization using FM. The results demonstrated that discs treated with MBIC_50_ of AKFAs showed few clumped matrices that were unequally distributed with lower surface adherence (Figure 7D). In contrast, *S. mutans* cells without treatment with AKFAs were adhered together with excess EPS matrix indicated by *S. mutans* cells with green florescence wrapped up with green extracellular adherence substances (Figure 7C).

### 3.5. Cytotoxicity of the Extracted AKFAs in Human Oral Fibroblast Cells

**Figure 8:**
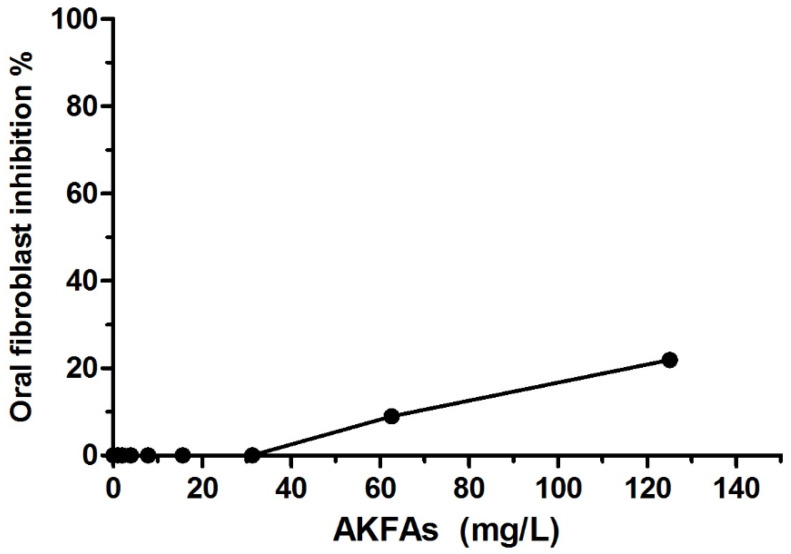
Minimum Cytotoxic effect of the AKFAs on human oral fibroblast cells.

In order to determine the cytotoxic effect of the AKFAs, human oral fibroblasts cells were treated with different concentrations of AKFAs, and the cell viability was measured. The toxicity of AKFAs to HEPM cells is shown in Figure 8. It was determined that even higher concentrations of AKFAs (125 mg L^−1^ and 62.5 mg L^−1^) have only a minor effect on cell viability. Moreover, the MBIC, and MIC_WI-EPS_ AKFA concentrations (31.3 mg L^−1^) produced no change in human oral fibroblasts cell viability.

## 4. Discussion

Dental caries are an important global health problem affecting all ages in both developed and developing countries [3]. *S. mutans* is considered the main etiological bacteria involved in the formation of dental caries and has the ability to establish cariogenic biofilms, as it is the major producer of EPS, as well as lipoteichoic acid [11]. Recently, a new trend in antimicrobial development has emerged to explore drugs that exclusively target the elaboration of the pathogen’s virulence factors rather than suppressing the pathogen itself. This approach can be seen as disarming the pathogen and rendering them harmless by making them susceptible to antibiotics or the human immune system [47]. Moreover, there is an urgent need to identify alternatives that selectively target biofilms produced by cariogenic bacteria without inducing oral dysbiosis, which could contribute to severe diseases [7]. Therefore, searching for natural therapeutic agents affecting *S. mutans* virulence factors is of significant concern. In spite of the demonstration of the large number of fungal metabolites that have been isolated and blocked virulence factors of many pathogens, up to now, the antivirulence activity of intracellular fungal fatty acids has not yet been elucidated [48,49].

In the present study, the endophytic fungus was isolated from the leaves of *Coriandrum sativum* and subjected to examination. FM observations revealed the ability of *Arthrographis kalrae* to store large amount of intracellular lipid globules. This result was in agreement with Paul et al. (2017), who reported that among all the endophytic fungi isolated from different plant segments, those isolated from the plant’s leaves showed higher lipid content [50]. Other previous studies detected large lipid globules within the hyphae of fungi [51,52]. The fungal isolate in this study was identified morphologically, microscopically and through ITS-based rDNA sequence comparison as *Arthrographis kalrae* MK967698.

Analysis of the isolated *Arthrographis kalrae* lipid contents revealed that among the nine fatty acids identified, five unsaturated fatty acids were detected with variable percentages composed of two major compounds, oleic (61%) and linolenic (23.9%). Minor compounds consisted of docosahexaenoic acid (4.6%), myristoleic (4.1%), and palmitoleic (0.2%).

Five of AKFAs—namely, palmitic, arachidic, stearic, oleic, and linolenic fatty acids—were previously detected in *Coriandrum sativum* plants [23]. In the same context, Kumar et al. (2013) found that endophytic *Colletotrichum truncatum*, *Nigrospora oryzae*, *Fusarium proliferatum*, *Guignardia cammillae*, *Alternaria destruens*, and *Chaetomium spendophytic* have a fatty acid constituents similar to those contained in their host, *Jatropha curcas* [53]. Close association between the entophytes and the plants results in genetic material exchange and subsequently apparent co-metabolism [51]. Also, Fakas et al. (2008), detected palmitic, stearic, oleic, linoleic, and linolenic acids in *Cunninghamella echinulate* fungus biomass [54]. Furthermore, *Kluyveromyces polysporus, Torulaspora delbrueckii, Yarrowia lipolytica, Candida* sp., *Trichosporon cutaneum, Rhodotorula glutinis,* and *Saccharomyces cerevisiae* were reported to produce oleic, palmitic, linoleic, stearic, arachidic, and palmitoleic fatty acids [55].

Moreover, the lipid profile of *Arthrographis kalrae* includes an omega-3 family member (docosahexaenoic acid polyunsaturated fatty acid, representing 4.6%). This finding was also supported by Francisco et al. (2017), who reported the ability of many filamentous fungi to release essential fatty acids belonging to the omega-3 family, especially some Mortierella species that showed the ability to produce docosahexaenoic and eicosapentaenoic acids [25].

Previous reports demonstrate the role of individual unsaturated fatty acids such as oleic acid that blocked the bacterial primary adhesion in a dose dependent manner and consequently suppressed the biofilm formation of *Staphylococcus aureus*, whereas the bacterial viable counts remained stable [56]. Also, linolenic acid was found to disrupt cell-to-cell communication, biofilm formation, and decreased the production of *Pseudomonas aeruginosa* virulence factor [57]. Furthermore, palmitoleic and myristoleic acids have the capability to inhibit the biofilm formation of *Acinetobacter baumannii* up to 38% and 24%, respectively, by inhibiting the *Acinetobacter baumannii* quorum sensing communication system [26]. Ten-undecynoic acid was reported to play an important role in Streptococcal oral biofilm prevention [3]. Some fatty acids were found to exhibit an anti-glucosyltransferase activity [58]. Herein, the effect of combined AKFAs on *S. mutans* biofilm was investigated. A potent antibiofilm activity against cariogenic *S. mutans* was detected. The antibiofilm activity of AKFAs might be attributed to their major constituents of unsaturated fatty acids or the synergistic effect exhibited by their major and minor constituents. 

In biofilms, the bacterial cells are embedded in an extracellular complex matrix, which accounts for more than 90% of the biofilm’s dry mass. These EPS consist of combination of bioactive molecules with high polarity such as proteins, polysaccharides, nucleic acids and lipids. It forms the scaffold for the biofilm three-dimensional structure in addition to facilitating the adhesion to surfaces and cohesion in the biofilm [59,60]. AKFAs were found to prevent the production of WI-EPS completely in *S. mutans* biofilm at the concentration of 31.3 mg L^−1^. Similarly, Giacaman et al. (2015) reported that monounsaturated (oleic), as well as polyunsaturated (linoleic) fatty acids significantly reduced EPS production in *S. mutans* biofilm. However, the concentrations that promote antibiofilm activity of each fatty acid alone was relatively high (2.8 g oleic and 2.7 g linoleic). These findings might justify the high antibiofilm activity of AKFAs in this study at an extremely low concentration (31.3 mg L^−1^). Furthermore, extraction of AKFAs from fungal biomass offers a cost- effective strategy that does not need further purification steps of the mixture of the fatty acids.

The results of this study showed an extremely positive correlation between biofilm formation and WI-EPS production by *S. mutans,* and this finding supports the idea that EPS production is a prerequisite for biofilm adhesion and is crucial in the first step of surface colonization [61].

Acidogenic *S. mutans* biofilm cells have the ability to ferment sugars especially sucrose, responsible for the production of strongly acidic environments (pH 4.5–5.5) and acid-degradation of nearby tooth enamel to start the onset of dental caries [11]. However, we demonstrate that acidogenesis produced by *S. mutans* biofilm can be mitigated. Fatty acids could act as micelles surrounding the individual oral bacterium and interfere with their adhesion to the tooth enamel in addition to inhibiting the bacterial metabolism and acid production [62]. Also, some of the acidogenesis-mitigation activity of AKFAs potentially accounted for oleic and linoleic activities [24].

Additionally, AKFAs activity was also conducted on S-H discs, which resemble the tooth surface in a scenario close to in vivo condition. SEM and FM observations revealed that AKFAs are able to repress biofilm formation and EPS production of *S. mutans* by disturbing the biofilm architecture, which results in a strong reduction of attached biofilm. Similar observation of biofilm changes was detected by SEM and FM, as demonstrated by Ansari et al. (2017)*,* who used self-aggregating peptide against *S. mutans* biofilm [40]. Nevertheless, AKFAs revealed significant inhibitory activity against the virulence factors of cariogenic *S. mutans*; they showed no bactericidal activity against the planktonic population of *S. mutans* at the concentration used. In the same context, Giacaman et al. (2014) reported that the unsaturated fatty acids have anti-metabolic activity against *S. mutans* virulence factors rather than antibacterial effects [24]. Furthermore, significant reduction in dental caries scores in rats was reported by incorporating fatty acids in a diet containing sugar [62].

Combining the antivirulence activities of AKFAs against *S. mutans* without exhibiting bactericidal or cytotoxic effect, support the potential use of AKFAs for prevention and treatment of dental caries. *Arthrographis kalrae* fatty acids isolated from coriander provide natural, safe and novel antibacterial strategy in antimicrobial development by targeting the microbial virulence factors instead of inhibiting the microorganisms. Further studies are needed for optimization of fatty acid production, as well as for developing a pharmaceutical formulation containing the fatty acids as anti-caries agents. Also, in vivo tests and clinical trials are recommended. All these studies will certainly contribute to the translation of isolated fatty acids to clinical development.

## 5. Conclusions

AKFAs can augment oral hygiene by targeting *S. mutans* virulence factors, particularly by inhibiting biofilm formation, EPS synthesis, and acidogenesis, which are the main causes of dental caries. Since AKFAs were able to disrupt the cariogenic effects of *S. mutans* in the presence of cariogenic sucrose using both the microtiter plate method and a tooth model, AKFAs have the potential to be used as anti-caries agent. 

## Figures and Tables

**Table 1:** Gas chromatography–mass spectrometry (GC-MS) analysis of *Arthrographis kalrae* fatty acids.

t_R_^a^	Compound ^b^	Peak Area (%)	Molecular Formula	Fragmentation Ion
	**Saturated fatty acids**	**5.8**		
17.8	Palmitic	1.2	C_16_H_32_O_2_	73, 93,256
25.8	Margaric	1.4	C_17_H_34_O_2_	74, 129, 270
35.7	Stearic	2.0	C_18_H_36_O_2_	74, 87, 298
39.3	Arachidic	1.2	C_20_H_40_O_2_	74, 91, 295
	**Unsaturated fatty acids**	**93.8**		
14.6	Myristoleic	4.1	C_14_H_26_O_2_	41,55, 226
17.2	Palmitoleic	0.2	C_16_H_30_O_2_	55, 263,268
29.2	Linolenic	23.9	C_18_H_30_O_2_	67, 81,292
32.7	Oleic	61.0	C_18_H_34_O_2_	264,55,44
42.9	Docosahexaenoic acid	4.6	C_22_H_32_O_2_	131, 91, 79
	**Total**	**99.6**		

^a^ t_R_, retention time (min). ^b^ Compounds are listed in the order of their elution. Identification based on comparison of mass spectra with WILEY/NIST mass spectral database.

**Table 2:** Effect of AKFAs on *S. mutans* biofilm formation and WI-EPS production. ***Significance level *p* < 0.0001. Tukey’s test: Means sharing the same superscript letter in the same column are not significantly different.

ACFAs (mg L^−1^)	Mean Biofilm Inhibitory % ± SD	Mean WIEPS Inhibitory % ± SD
**0.00**	0.00 ^f^ ± 0.00	0.00 ^f^ ± 0.00
**1**	0.00 ^f^ ± 0.00	0.00 ^f^ ± 0.00
**2**	15.11 ^e^ ± 0.19	15.84 ^e^ ± 0.18
**3.9**	32.98 ^d^ ± 0.85	34.92 ^d^ ± 0.27
**7.8**	62.89 ^c^ ± 0.64	64.78 ^c^ ± 0.57
**15.6**	82.27 ^b^ ± 0.32	83.89 ^b^ ± 0.7
**31.3**	100.0 ^a^ ± 0.00	100.0 ^a^ ± 0.00
**62.5**	100.0 ^a^ ± 0.00	100.0 ^a^ ± 0.00
**125**	100.0 ^a^ ± 0.00	100.0 ^a^ ± 0.00
	MBIC_50_ = 6.1 mg L^−1^	M_EPS_IC_50_ = 5.9 mg L^−1^
	MBIC_100_ = 31.3 mg L^−1^	M_EPS_IC_100_ = 31.3 mg L^−1^
	*p* < 0.0001***	*p* < 0.0001***

## Supplementary Materials

The following are available online at https://www.mdpi.com/2218-273X/10/5/811/s1, Figure S1. Untreated biofilm *S. mutans* (A) compared to inhibitory effect of MBIC of AKFAs on biofilm viable cells (B) cultured on mitis salivarius bacitracin agar medium; Figure S2. (A) Acid mitigation effect of MBIC of AKFAs on *S. mutans* biofilm compared to untreated *S. mutans* biofilm (B).
