# Supplementary material for: Hindering of Cariogenic Streptococcus mutans Biofilm by Fatty Acid Array Derived from an Endophytic Arthrographis kalrae Strain
Source: Biomolecules. 2020 May 25;10(5):811. doi: 10.3390/biom10050811 (PMC7277960; doi:10.3390/biom10050811)

Supplementary Materials

Hindering of Cariogenic *Streptococcus mutans* Biofilm by Fatty Acid Array Derived from an Endophytic *Arthrographis kalrae* Strain

Marwa M. Abdel-Aziz[1], Tamer M.Emam[2], and Marwa M. Raafat[3,*]

[1]Regional Center for Mycology and Biotechnology (RCMB), Al-Azhar University, Cairo 11651, Egypt; marwa2rcmb@yahoo.com

[2] Microbiology Department, Desert Research Center (DRC), Cairo 11753, Egypt; tameremam7@gmail.com

[3]Microbiology and Immunology Department, Faculty of Pharmaceutical Sciences and Pharmaceutical Industries, Future University in Egypt (FUE), Cairo 11835, Egypt. marwa.mahmoud@fue.edu.eg

* Correspondence: marwa.mahmoud@fue.edu.eg; Tel.: +201001860189

Figure 1. Untreated biofilm *S. mutans* (**A**) compared to inhibitory effect of MBIC of AKFAs on biofilm viable cells (**B**) cultured on mitis salivarius bacitracin agar medium.

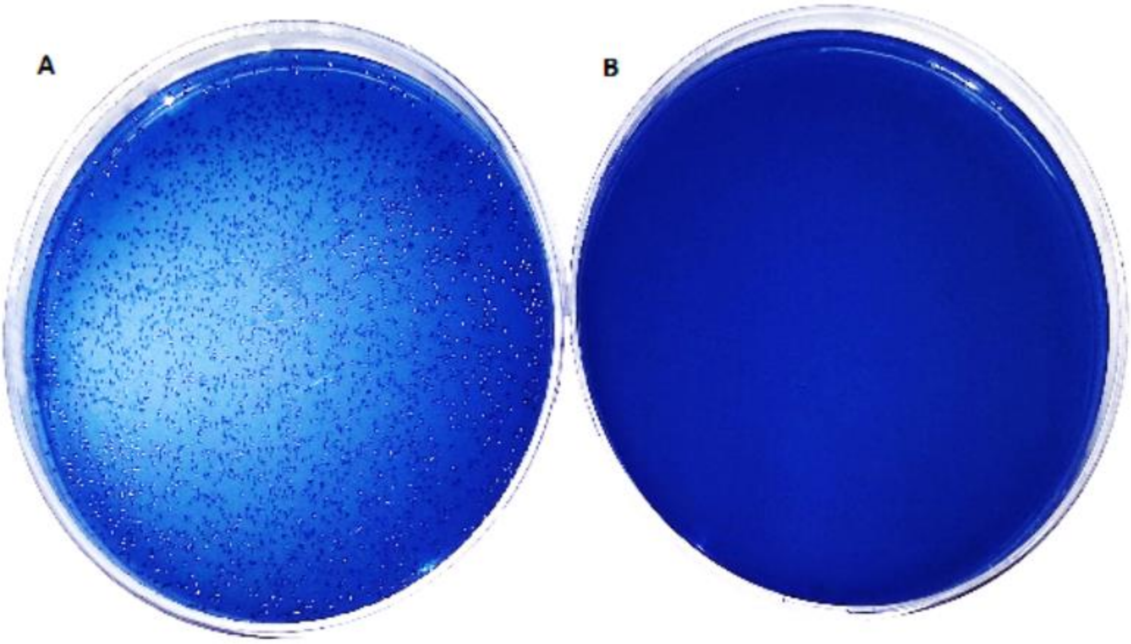

Figure S2. (**A**) Acid mitigation effect of MBIC of AKFAs on *S. mutans* biofilm compared to untreated *S. mutans* biofilm (**B**).

.

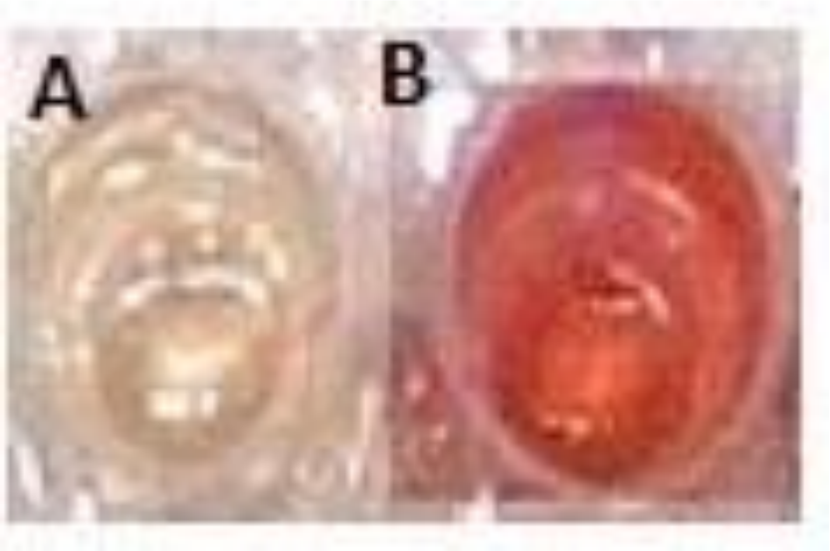

Supplement: Supplementary file 1 [file biomolecules-10-00811-s001.pdf]
